# A genome-wide scanning and fine mapping study of COGA data

**DOI:** 10.1186/1471-2156-6-S1-S30

**Published:** 2005-12-30

**Authors:** Hsin-Chou Yang, Chien-Ching Chang, Chin-Yu Lin, Chun-Liang Chen, Chin-Yu Lin, Cathy SJ Fann

**Affiliations:** 1 Institute of Biomedical Sciences, Academia Sinica, Taipei, Taiwan; 2 Institute of Public Health, Yang-Ming University, Taipei, Taiwan

## Abstract

A thorough genetic mapping study was performed to identify predisposing genes for alcoholism dependence using the Collaborative Study on the Genetics of Alcoholism (COGA) data. The procedure comprised whole-genome linkage and confirmation analyses, single locus and haplotype fine mapping analyses, and gene × environment haplotype regression. Stratified analysis was considered to reduce the ethnic heterogeneity and simultaneously family-based and case-control study designs were applied to detect potential genetic signals. By using different methods and markers, we found high linkage signals at D1S225 (253.7 cM), D1S547 (279.2 cM), D2S1356 (64.6 cM), and D7S2846 (56.8 cM) with nonparametric linkage scores of 3.92, 4.10, 4.44, and 3.55, respectively. We also conducted haplotype and odds ratio analyses, where the response was the dichotomous status of alcohol dependence, explanatory variables were the inferred individual haplotypes and the three statistically significant covariates were age, gender, and max drink (the maximum number of drinks consumed in a 24-hr period). The final model identified important AD-related haplotypes within a candidate region of *NRXN1 *at 2p21 and a few others in the inter-gene regions. The relative magnitude of risks to the identified risky/protective haplotypes was elucidated.

## Background

Alcohol dependence (AD) is a complex disorder accompanying familial aggregation and etiological heterogeneity. The development of AD involves genetic and environmental components as well as gene × gene and gene × environmental interactions. Due to these factors, results from different studies often diverge [1].

Owing to the advancement of biotechnology, enormous numbers of short tandem repeat polymorphisms (STRPs) and single-nucleotide polymorphisms are available to help the process of gene mapping. In this report, STRP and SNP markers were integrated and a five-stage procedure was designed to identify the putative AD loci and to elucidate the genotype-phenotype-covariate relationship. Different methodologies (linkage analysis, association fine mapping, haplotype inference, and regression model) were considered for statistical analyses, different populations (whole, non-Black, and non-White populations) for heterogeneity issues, different types of markers (STRPs and SNPs) for linkage mapping, different densities of SNPs (Illumina and Affymetrix) for association study, and different data structures (family data and case-control data) for study design to yield reliable conclusions.

## Methods

### Data description

From the COGA ascertainment criteria, the numbers of total patients, pure unaffected individuals, and others were 643 (39.84%), 285 (17.66%), and 686 (42.50%), respectively. The category "others" was considered as "unknown" throughout our analyses. On average, 60% of parents' genotypes were available.

In total, 315 STRPs, 4,720 Illumina SNPs, and 11,120 Affymetrix SNPs on the 22 autosomal chromosomes with average spacing of 11.53 cM, 0.75 cM, and 0.32 cM were considered. The genetic map was provided by the Genetic Analysis Workshop 14 (GAW14) working group.

Ethnic heterogeneity was considered by stratifying the studied families as pure "non-Black" and "non-White" families, i.e., families where none of the members were from the Black population and vice versa. The non-Black population contained 1,300 individuals from 119 families and non-White families contained 247 individuals from 19 families. Other families were not included in this report. In addition to family data, founders from each family were selected for case-control data that contained 505 individuals with 52 affected (cases), 127 unaffected (controls) and 326 individuals with other phenotypes.

### Statistical methods

To explore the phenotype × genotype relationship and locate the AD predisposing genes, we carried out a five-stage procedure. The first stage was designed to search the potential candidate regions by considering a genome-wide linkage analysis using the STRP markers. GENEHUNTER [2] and SIMWALK2 [3] were employed to conduct multipoint nonparametric linkage (NPL) analysis, using the 'all' scoring function. Five evenly spaced positions scanning between markers were used. The allele frequencies were provided by GAW14 working group. A chromosome region with an NPL score greater than 3 was identified as "highly linked with AD".

The second stage used denser SNP markers to confirm linkage results obtained in the first stage. On the basis of the NPL scores from the first stage, a candidate region was defined to be a segment in which all NPL scores exceeded 1 and the maximal NPL score exceeded 3. In the candidate regions, SIMWALK2 [3] was carried out for multipoint linkage analyses using Illumina and Affymetrix SNP markers. The results were compared with those obtained from the first stage.

In the third stage, association analyses were conducted using SNPs to further narrow the candidate region. Transmission disequilibrium tests were performed by using PDT [4] and FBAT [5] for family data, and linkage disequilibrium tests (allele-based association test [6]) were used for case-control data.

In the fourth stage, anchor markers were selected on the basis of results from the third stage. HAPLOVIEW [7] was used to construct haplotype blocks and select tag SNPs in the region determined by anchors and nearby markers. Inferences on genotype-phenotype relationship were drawn by results obtaining from haplotype analysis using SIMWALK2 [3] for family data and PHASE2 [8] for case-control data.

**Figure 1:**
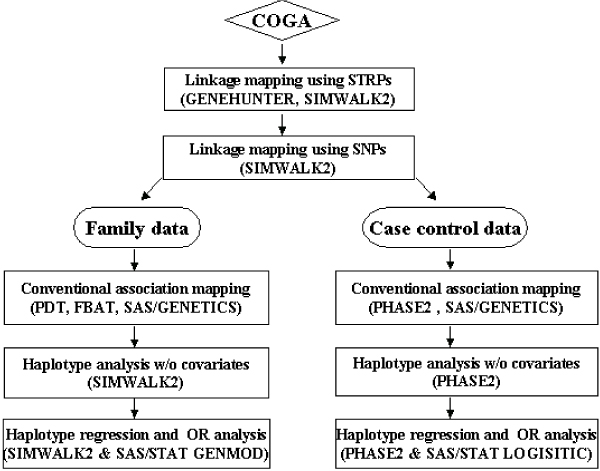
The flow chart of statistical analyses.

In the fifth stage, the relationships between genotype, phenotype, and covariates underlying the complex alcoholism etiology was further explored. The individual haplotypes were inferred based on results obtained from SIMWALK2 [3] for family data and PHASE2 [8] for case-control data. The inferred individual haplotypes and important demographic variables, risk factors, and other phenotypes were modelled simultaneously with the explanatory variables in the regression models. For family-based analysis, the generalized estimating equation approach using the procedure GENMOD of the package SAS/STAT [9] was applied; for case control analysis, an unconditional logistic regression using the procedure LOGISTIC of the package SAS/STAT [9] was applied. The flow chart of statistical analyses is shown in Figure 1.

## Results

**Figure 2:**
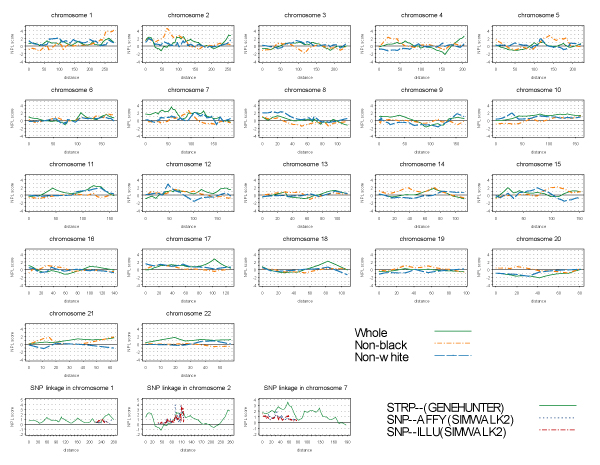
NPL scores of genome-wide linkage analysis.

A genome-wide multipoint linkage analysis for the 22 pairs of autosomal chromosomes based on the 315-STRP markers using GENEHUNTER [2] was conducted. Figure 2 (the green solid line) shows that NPL score > 3 only occurs on chromosome 7 and the highest NPL score (3.54866) is located at D7S2846.

To reduce false-positives due to population heterogeneity, stratified analyses by selecting non-Black and non-White subpopulations from the whole population was conducted. Whole-genome linkage mapping with STRP markers was applied to these two subpopulations and yielded rather different results compared with the whole population. The results are shown in Figure 2. For the non-White population (the blue dashed line), no NPL score was found to be larger than 3, which might be due to small sample size in this subpopulation. For the non-Black population (the orange dot-point line), the NPL scores for D1S225, D1S547, and D2S1356 are 3.91886, 4.10389, and 4.43759, respectively. Results obtained from GENEHUNTER [2] and SIMWALK2 [3] are quite consistent (results not shown).

In the second stage, we conducted SNPs linkage analysis to confirm the STRP linkage results of chromosomes 1, 2, and 7 found in the first stage. The three candidate regions determined by the mentioned criteria were D1S518-D1S547, D2S320-D2S436, and D7S1790-D7S665. In these three candidate regions, the Linkage III Panel of SNPs of Illumina consists of 38, 151, and 103 SNPs and the inter-marker distances are 0.99, 0.53, and 0.74 cM in average. The GeneChip Mapping 10 K Array marker set of SNPs of Affymetrix consists of 113, 344, and 238 SNPs and the average distances between markers are 0.47, 0.23, and 0.30 cM. The results confirm the previous linkage results and find significant Illumina and Affymetrix SNPs with NPL scores > 3 on chromosome 2 as shown in Figure 1; however, the NPL curves are not the same as the curve obtained from STRPs previously.

In the third stage, further fine mapping was pursued to narrow down the candidate regions using association tests. Based on family-based transmission disequilibrium tests (PDT [4] and FBAT [5]) and case-control linkage disequilibrium tests (allele-based test [6]), the SNPs associated with AD (*p*-value < 0.01) without correcting multiple tests are shown in Table 1, where *p*-values are transformed by taking -log_10_.

In the fourth stage, we selected the most significant SNPs to be anchor markers based on Table 1 and preceded with finding haplotype blocks and tag SNPs in the region. Only the block closest to the anchor marker was used to conduct haplotype analysis without adjusting covariates. However, no significant haplotypes were found.

In the fifth stage, haplotype regression analyses considering three significant covariates (age, gender, and max drink) were conducted, which were selected in preliminary analysis. Results of adjusted odds ratio are summarized in Table 2. On chromosome 1, no significant haplotypes were found. On chromosome 2, haplotypes 11 and 12 constituted by SNPs rs977744 and tsc0794923 yield ORs 0.001 and 0.009 and 95% CIs (<0.001, 0.207) and (0.001, 0.171), respectively, and show strong protective effects; haplotype 2222, comprising SNPs tsc0063067, tsc0059588, tsc0043992, and tsc1473501 at gene *NRXN1*, yields an OR of 0.65 with 95% CI (0.45, 0.93). On chromosome 7, haplotype 111 from SNPs tsc0018713, tsc0018712, and tsc0593964 is a risk haplotype with an OR of 2.13 and corresponding 95% CI (1.09, 4.15).

## Discussion

In summary, some potential candidate regions on chromosomes 1, 2, and 7 linked with AD susceptibility loci were found. These findings are consistent with previous reports [10,11]. Moreover, association and haplotype analyses further narrowed the candidate region. On chromosome 2, a haplotype within the intronic region of gene *NRXN1 *related to polymorphic cell surface proteins was identified, as well as two strongly protective haplotypes in inter-gene regions. On chromosome 7, one moderately risky haplotype in an inter-gene region was identified. These results should be useful to biologists for the advanced study of functional cloning.

The linkage scans based on three different marker sets were compared. The curves of NPL scores based on two SNP sets are quite similar; however, the SNP scans and STRP scan show somewhat inconsistent results on different chromosomes. On chromosome 2, SNP linkage scan confirms STRP scan and yields more and higher linkage signals in the same region. In other candidate regions, SNP scans fail to identify any important SNPs, probably due to their lower information content. We also compared the results from three association tests and found many different significant SNPs based on family-based and case control association tests. The differences were probably due to the different samples used in the analyses and information extracted from transmission and linkage disequilibrium tests.

Our five-stage gene mapping procedure is elaborate though incomplete. Other analytical strategies, such as quantitative trait analysis, will provide complementary information to further dissect the etiology of AD.

## Abbreviations

AD: Alcohol dependence

COGA: Collaborative Study on the Genetics of Alcoholism

GAW: Genetic Analysis Workshop

NPL: Nonparametric linkage

SNP: Single-nucleotide polymorphism

STRP: Short tandem repeat polymorphism

## Authors' contributions

H-CY conceived the statistical analysis scheme, coordinated the project and drafted the manuscript. CSJF contributed to the discussion and preparation of the final manuscript. Other members carried out the data management, statistical analysis and technique assistance. All authors have approved the final manuscript.

## Figures and Tables

**Table 1:** Summary of results of transmission and linkage disequilibrium tests

Chr.	SNP	Position	PDT	FBAT	Allele
1	rs1906255	182.8			2.75
	tsc0073840	204.8			2.02
	tsc0982091	212.1	2.41		
	tsc1100744	213.3			2.49
	rs908857	214.1	2.37		
	tsc0054439	215.3			2.13
	tsc1512021	219.3			2.29
	tsc0040851	219.6			2.42
	tsc0056649	224.8			2.60
	tsc0576563	225.6			2.25
	tsc1001020	226.4			3.42
	tsc0059489	226.7		2.88	
	tsc0990050	229.3			4.22
	tsc0998408	234.7	2.01		
	tsc0272628	238.9			2.68
	tsc0832886	248.6			2.99
	rs946001	257.0	2.41		
	tsc0046578	260.0		2.99	

2	tsc1457260	40.79			2.04
	tsc0275086	45.06	2.49		
	rs2033654	47.60	2.30	2.09	
	tsc1518799	55.32		2.69	
	rs927087	56.10	2.25		
	tsc0273405	56.65			2.81
	tsc0764714	59.83	2.00		
	rs726548	66.90		2.00	
	tsc0788448	71.22			2.15
	tsc0043992	75.99	3.15		
	tsc0270239	80.94		2.17	
	rs2008312	88.20			2.46
	rs977744	102.1			3.93
	tsc0794923	102.5	2.13		

7	tsc0050391	5.21			2.02
	tsc1110728	13.65		2.02	
	tsc0847689	17.81			2.88
	tsc0064419	18.04			2.60
	tsc0247731	20.41		2.38	
	tsc1061178	29.05		2.08	
	rs957960	29.30			2.02
	tsc0331830	33.94		2.15	
	tsc0050450	35.30			2.07
	rs798646	37.90			2.14
	tsc0593964	42.62	2.72	3.48	
	tsc0042959	44.49		2.69	
	tsc0051325	47.56	2.17	2.24	
	tsc0054307	47.85	2.17	2.24	
	tsc0462262	61.4514			2.45
	tsc1241245	62.6748	2.14		
	tsc0893346	63.6850			2.37
	tsc0065973	70.5578			2.00
	tsc0260324	73.8253			2.13
	tsc0525473	75.0415			2.26
	tsc1407569	75.6400			4.75

**Table 2:** Summary of results of haplotype analysis with covariates

Study design	Chr	SNPs in haplotype	Significant haplotypes	OR (95% CI)
Family-based	1	tsc0046578^a^-tsc0938317	---^b^	---
Family-based	2	tsc0063067-tsc0059588-tsc0043992-tsc1473501^a^	2222	0.65 (0.45, 0.93)
Family-based	7	tsc0018713-tsc0018712-tsc0593964^a^	111	2.13 (1.09, 4.15)
Case control	1	tsc0949090-tsc0755351-tsc0990050^a^	---	---
Case control	2	rs977744^a^-tsc0794923	11	0.001 (<0.001, 0.207)
			12	0.009 (0.001, 0.171)
Case control	7	tsc0272090-tsc1407569^a^	---	---

^a^Anchor marker

^b^---, no significant haplotypes were identified.
